# Dysregulation of acyl carnitines, pentose phosphate pathway and arginine and ornithine metabolism are associated with decline in intrinsic capacity in Chinese older adults

**DOI:** 10.1007/s40520-023-02654-x

**Published:** 2024-02-12

**Authors:** Yiming Pan, Yun Li, Jagadish K. Chhetri, Pan Liu, Bowen Li, Zuyun Liu, Guanghou Shui, Lina Ma

**Affiliations:** 1 Department of Geriatrics, Xuanwu Hospital, Capital Medical University, National Research Center for Geriatric Medicine, 45 Changchun Street, Beijing, 100053 China; 2 Department of Neurology and Neurobiology, Xuanwu Hospital of Capital Medical University, Beijing, 100053 China; 3 National Clinical Research Center for Geriatric Diseases, Xuanwu Hospital of Capital Medical University, Beijing, 100053 China; 4 State Key Laboratory of Molecular Developmental Biology, Institute of Genetics and Developmental Biology, Chinese Academy of Sciences, Beijing, 100101 China; 5 Center for Clinical Big Data and Analytics, Second Affiliated Hospital and Department of Big Data in Health Science, School of Public Health, The Key Laboratory of Intelligent Preventive Medicine of Zhejiang Province, Zhejiang University School of Medicine, Hangzhou, 310058 Zhejiang China; 6 LipidALL Technologies Company Limited, Changzhou, 213022 Jiangsu China

**Keywords:** Functional decline, Aging, Intrinsic capacity, Metabolomics

## Abstract

**Background:**

Intrinsic capacity is the combination of individual physical and mental abilities, reflecting the aging degree of the older adults. However, the mechanisms and metabolic characteristics of the decline in intrinsic capacity are still unclear.

**Aims:**

To identify metabolic signatures and associated pathways of decline in intrinsic capacity based on the metabolite features.

**Methods:**

We recruited 70 participants aged 77.19 ± 8.31 years. The five domains of intrinsic capacity were assessed by Short Physical Performance Battery (for mobility), Montreal cognition assessment (for cognition), 30-Item Geriatric Depression Scale (for psychology), self-reported hearing/visual impairment (for sensory) and Nutritional risk screening (for vitality), respectively. The serum samples of participants were analyzed by liquid chromatography-mass spectrometry-based metabolomics, followed by metabolite set enrichment analysis and metabolic pathway analysis.

**Results:**

There were 50 participants with a decline in intrinsic capacity in at least one of the domains. A total of 349 metabolites were identified from their serum samples. Overall, 24 differential metabolites, 5 metabolite sets and 13 pathways were associated with the decline in intrinsic capacity.

**Discussion:**

Our results indicated that decline in intrinsic capacity had unique metabolomic profiles.

**Conclusion:**

The specific change of acyl carnitines was observed to be a feature of decline in intrinsic capacity. Dysregulation of the pentose phosphate pathway and of arginine and ornithine metabolism was strongly associated with the decline in intrinsic capacity.

**Supplementary Information:**

The online version contains supplementary material available at 10.1007/s40520-023-02654-x.

## Introduction

With the improvement of health care and socioeconomic conditions, human life expectancy continues to rise. According to the World Health Organization (WHO) data, one in six people in the world will be aged 60 years or over by 2030 [1]. However, longevity is not consistent with healthy life expectancy. The aging of the global population is accompanied by the decline in daily life abilities and stress ability of older adults, and a huge burden of disease [2]. In 2015, the WHO published *World Report on Ageing and Health*, which defines healthy aging as the process of developing and maintaining the functional ability that enables well-being in old age [3]. Intrinsic capacity (IC), defined as the sum of all physical and mental capabilities of an individual, is one of the key elements of functional ability. Hence, IC can be considered as a reliable indicator of healthy aging in older adults. IC is proposed to be composed of five major domains of functions, including mobility, cognition, psychology, sensory, and vitality.

As a new medical paradigm for evaluating the health of older adults, there is an increasing interest on this new entity among aging researchers and clinicians. However, past studies on IC are largely focused on validation and predicting negative outcomes in older adults. Studies have shown decline in IC to be associated to frailty, disability, and decline in instrumental activities of daily living and activities of daily living in older adults [4, 5]. Nonetheless, research on the underlying biological mechanism related to IC is still in its infancy, and the metabolic changes of the decline in IC are still unknown. Increasingly optimized metabolomics techniques allow reliable identification and quantification of metabolites from biological samples. The application of metabolomics helps to deepen understanding of diseases, which is of great value for the explanation of the underlying mechanism of the decline in IC.

In our study, we applied metabolomics methods to explore IC-related metabolites and pathway changes, and compared and analyzed the similarities, differences and connections of metabolic characteristics among various domains of IC. We hoped to preliminarily characterize the metabolomics signatures of IC decline.

## Materials and methods

### Study design and participants

**Fig. 1:**
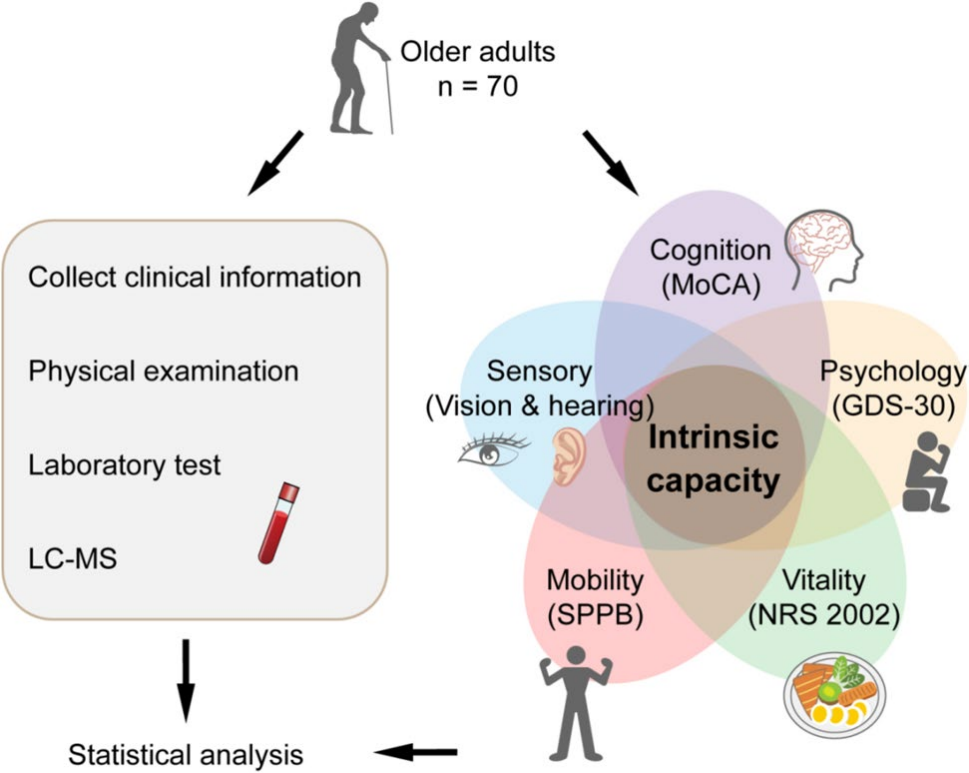
Schematic diagram of the study protocol. Gather clinical information, perform intrinsic capacity (IC) assessment on all participants, and collect blood for untargeted metabolomics analysis and laboratory tests. Abbreviations: LC–MS, liquid chromatography-mass spectrometry; SPPB, short physical performance battery; MoCA, Montreal cognition assessment; GDS, geriatric depression scale; NRS, nutritional risk screening

In this cross-sectional study, we enrolled 70 adults aged 61–88 years, who received regular health check-ups at the Geriatrics Department of Xuanwu Hospital, Capital Medical University. The study design is shown in Fig. 1. People with acute infectious diseases, single or multiple organ failure, malignant tumors, unstable vital signs, or unable to cooperate with the assessment were excluded. All participants have not received general anesthesia and have not used psychotropic drugs in the past three months. Basic information of all participants (including gender, age, marriage, education, smoking and drinking) was collected and a detailed medical history (including chronic disease, medication) was taken. All participants went through routine physical examination, laboratory tests, and IC assessment (Figure S1).

All participants signed the informed consent following the Declaration of Helsinki and the study was approved by the ethical review board of Xuanwu Hospital Capital Medical University.

### IC assessment

IC assessment was performed according to the recommendations released by the WHO. We used Short physical performance battery (SPPB) [6] for mobility; Montreal cognition assessment (MoCA) [7] for cognition; 30-item Geriatric depression scale (GDS-30) [8] for psychology; self-reported hearing or visual impairment for sensory; and Nutritional risk screening (NRS-2002) [9] for vitality. The cut-off points of each assessment tool are shown in Table S1. We rated the scores of each domain as 0 (worst performance), 1 (medium performance) and 2 (best performance) points. The overall IC score was the sum of the rated scores of the five domains, ranging from 0 (worst IC) to 10 (best IC). Participants with total IC score ≤ 9 were grouped as “IC decline”; participants with IC score = 10 were grouped as “non-decline in IC”.

### Sample size calculation

As an exploratory study, since differential metabolites were unknown at the outset, it was difficult to estimate metabolite changes in advance and almost impossible to estimate sample size [10]. We could only estimate the sample size based on experience. Previous studies have shown that metabolomics studies are credible enough if the sample size is greater than 20 cases [11]. Therefore, we included a total of 70 cases based on enrollment status.

### Blood sampling and determination

All participants were required to quit smoking and alcohol and fast for at least 8 h before blood sample collection. Blood was collected from the median vein of the elbow and sent to our hospital’s laboratory for testing within two hours. Cholesterol (reference range 3.2–5.7 mmol/L), triglycerides (reference range 0.45–2.25 mmol/L), high-density lipoprotein (reference range 1.08–1.91 mmol/L), low-density lipoprotein (reference range 2.08–3.12 mmol/L), uric acid (reference range 155–428 umol/L), creatinine (reference range 18–104 umol/L), blood urea nitrogen (reference range 1.7–8.3 mmol/L), albumin (reference range 35–55 mg/L), prealbumin (reference range 170–420 mg/L), aspartate aminotransferase (reference range 8–40 IU/L), alanine aminotransferase (reference range 5–40 IU/L), C reactive protein (reference range 1–8 mg/L), D-dimer (reference range 0.01–0.5 ug/mL), and fasting plasma glucose (reference range 3.9–6.1 mmol/L) were determined by the Hitachi 7600 automatic analyzer (Mannheim, Germany). Hemoglobin A1c (reference range 4–6%) was determined by the Biorad Diomat high-pressure liquid chromatography analyzer (Philadelphia, PA). Hemoglobin (reference range 120–160 g/L) were determined by the SYSMEX XE-2100 Hematology Analyzer (Tokyo, Japan).

### Metabolomics analysis[12]

#### Serum sample collection

Centrifugation (3000 rpm × 5 min) of the blood sample was performed within 2 h of sample collection, and the serum was stored at -80℃ refrigerator.

#### Reagents

Water was purified using ultrapure water preparation system; liquid chromatography-mass spectrometry (LC–MS) grade acetonitrile and methanol were purchased from Merck (Germany); Ultra-performance liquid chromatography grade formic acid was obtained from Sigma (Germany). All internal standard references were purchased from Cambridge Isotope Laboratories (U.S), including Phenylalanine-D8, Tryptophan-D8, Isoleucine-D10, Asparagine-13C4, Methionine-D3, Valine-D8, Proline-D7, Alanine-D4, Glycine-D2, Serine-D3, Glutamate-D5, Aspartate-D3, Arginine-D7, Glutamine-D5, Lysine-D9, Histidine-D5, D13-choline, (13C,15N3)-Uric acid, (15N)4-Inosine, D5-Benzoic acid, D11-Betaine and P-cresol sulfate-D7. Quality control (QC) samples were prepared by mixing all serum samples according to identical steps as the actual serum samples.

#### Metabolome extraction

Add 50 µL of plasma with 200 µL of ice-cold methanol, incubate for 30 min at 1500 rpm and 4 ℃, centrifuge for 10 min at 12,000 rpm and 4 ℃, remove the supernate into a clean 1.5 ml centrifuge tube, and dry using SpeedVac. The dried extracts were redissolved with 1% acetonitrile in water, and upper layer liquids were collected for LC–MS analysis.

#### Instruments

The column ACQUITY UPLC HSS T3 1.8 µm, 2.1 × 100 mm columns (Waters, Dublin, Ireland) was adopted into the present study. Ultra-performance Liquid Chromatography (Agilent 1290 II, Agilent Technologies, Germany) coupled to Quadrupole-TOF MS (5600 Triple TOF Plus, AB SCIEX, Singapore) was applied to acquire metabolome data.

#### Data processing

Data acquisition and processing were performed using Analyst® TF 1.7.1 Software (AB Sciex, Concord, ON, Canada). All detected ions were extracted using MarkerView 1.3 in the format of two-dimensional matrix, including mass to charge ratio (m/z), retention time and peak areas, and isotopic peaks were filtered. Raw MS data were extracted with the parameter setting as retention time tolerance less than 0.1 min during alignment. Each metabolite across the whole batch followed this retention time rule, otherwise it would be removed. PeakView 2.2 was applied to extract MS/MS data and to perform a comparison with the Metabolites database, HMDB, and standard references to annotate ion ID.

#### Statistical analysis

Continuous variables were presented as the mean ± standard deviation, and categorical variables were shown as frequencies (percentages). The difference between the two groups was compared by independent t-test for continuous variables, and by chi-square test for categorical variables. Spearman correlation analysis and linear regression were used to identify associations between differential metabolites and changes of IC, and differences were considered significant with a *p* value < 0.05. The above analyses were performed with SPSS (Armonk, NY, USA, version 26.0) and GraphPad Prism 8.2.1 (GraphPad Software, San Diego, CA, USA).

For metabolomics data, zero or missing values were replaced by 1/5th of the minimum positive value for each variable. Metaboanalyst 5.0 (https://www.metaboanalyst.ca/) was used for fold Change (FC) analysis, principal component analysis (PCA), orthogonal partial least squares discriminant analysis (OPLS-DA), metabolite set enrichment analysis (MSEA) and metabolic pathway analysis (MetPA).

## Result

**Fig. 2:**
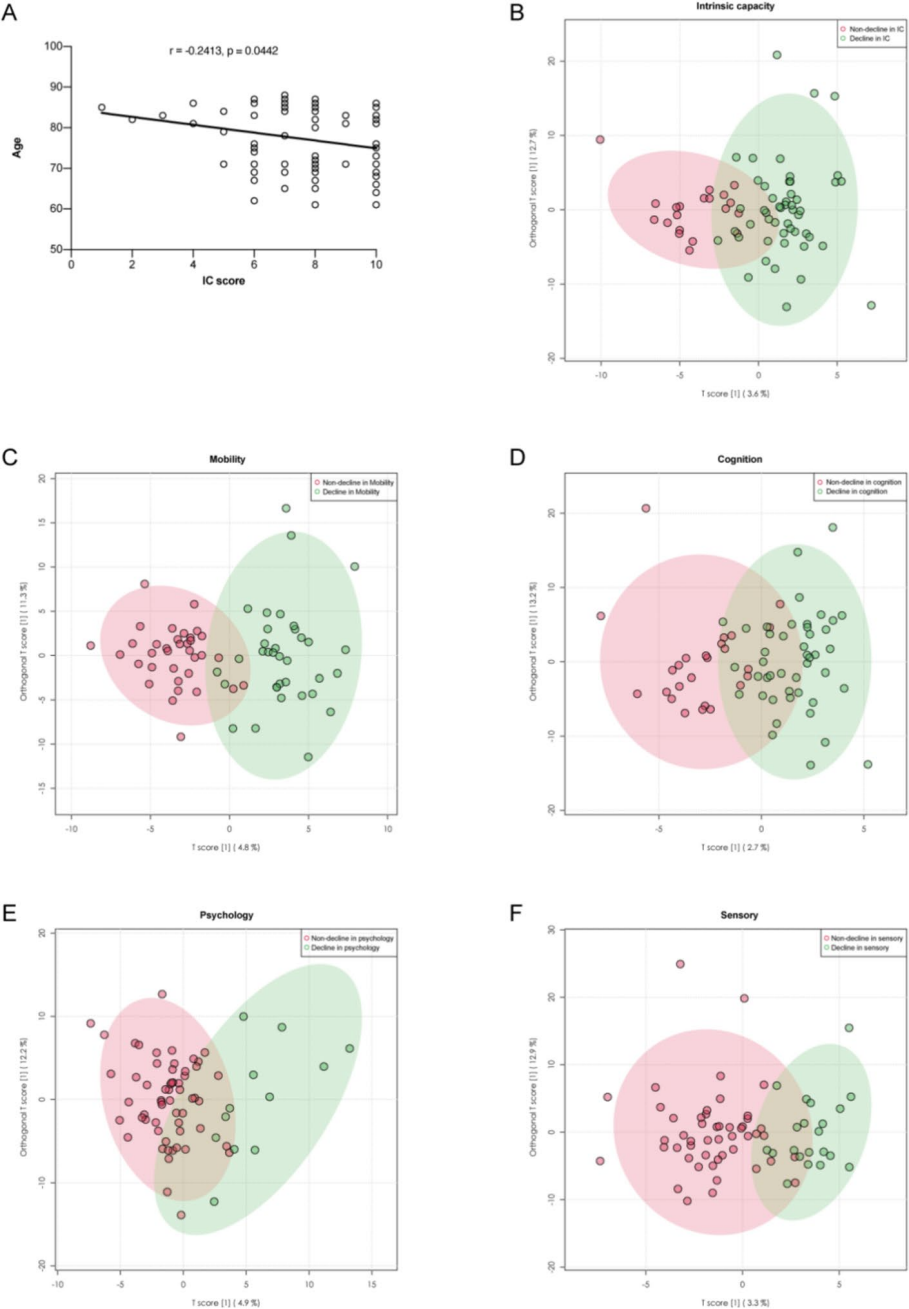
**A** Pearson correlation analysis between intrinsic capacity (IC) score and age. **B** OPLS-DA score plots of the decline in IC group and control. **C** OPLS-DA score plots of the decline in mobility group and control. **D** OPLS-DA score plots of the decline in cognition group and control. **E** OPLS-DA score plots of the decline in psychology group and control. **F** OPLS-DA score plots of the decline in sensory group and control. *IC* intrinsic capacity, *OPLS-DA* orthogonal partial least squares discriminant analysis

The mean age of the participants was 77.19 ± 8.31 years and 44 (62.9%) were male. The average IC score of 70 participants was 7.66 ± 2.07, of which 50 were categorized in the decline in IC group (with IC score 6.72 ± 1.70). While comparing general characteristics, D-dimer level was statistically different among different IC groups (Table 1). The results of Pearson correlation analysis showed that IC declined with age (Fig. 2A). No statistical differences (*p* value = 0.121) in IC scores were observed between males (7.98 ± 1.80) and females (7.12 ± 2.41). The number (and percentage) of participants with declines in mobility, cognition, psychology, sensory and vitality were 35 (50.0%), 45 (64.3%), 12 (17.1%), 21 (30.0%) and 2 (2.9%), respectively.

**Table 1:** Characteristics of the non-intrinsic capacity (IC) decline and IC decline group

Variables		Non-IC decline (*n* = 20)	IC decline (*n* = 50)	*p* value
General information	Age (year)	74.25 ± 8.51	78.36 ± 8.02	0.072
	Male (n, %)	14 (70.0)	30 (60.0)	0.434
	Marriage			
	Married (*n*, %)	17 (85.0)	35 (15.0)	0.195
	Widowed (*n*, %)	35 (70.0)	15 (30.0)	
	High school and above (*n*, %)	17 (85.0)	25 (50.0)	0.023
	Smoking (*n*, %)	4 (20.0)	6 (12.0)	0.429
	Drinking (*n*, %)	3 (15.0)	10 (20.0)	0.554
Medical history	Number of chronic diseases	5.65 ± 2.72	5.10 ± 2.71	0.452
	Number of drugs	5.80 ± 3.33	5.94 ± 4.01	0.886
Physical examination	Body mass index (kg/m^2^)	24.99 ± 2.89	24.65 ± 4.54	0.712
	Systolic blood pressure (mmHg)	142.30 ± 18.58	137.96 ± 20.04	0.394
	Diastolic blood pressure (mmHg)	73.80 ± 14.09	72.78 ± 10.66	0.772
IC assessment	SPPB	11.05 ± 0.94	6.78 ± 3.85	< 0.001
	MoCA	26.95 ± 1.88	20.10 ± 5.25	< 0.001
	GDS-30	3.75 ± 2.69	6.22 ± 4.89	0.037
	Visual impairment (*n*, %)	0 (0)	13 (26.0)	0.012
	Hearing impairment (*n*, %)	0 (0)	15 (30.0)	0.006
	NRS-2002	0.75 ± 0.85	0.74 ± 1.12	0.968
	IC score	10.00 ± 0.00	6.72 ± 1.70	< 0.001
Laboratory tests	D-dimer (ug/mL)	0.41 ± 0.21	0.88 ± 1.29	0.019
	C reactive protein (mg/L)	2.75 ± 1.71	5.32 ± 10.46	0.136
	Hemoglobin (g/L)	136.90 ± 13.19	129.89 ± 18.89	0.089
	Albumin (mg/L)	39.05 ± 0.94	38.09 ± 2.11	0.191
	Prealbumin (mg/L)	238.35 ± 43.04	221.89 ± 61.055	0.278
	Aspartate aminotransferase (IU/L)	24.60 ± 12.18	23.70 ± 7.48	0.762
	Alanine aminotransferase (IU/L)	20.40 ± 12.32	17.81 ± 11.70	0.429
	Creatinine (umol/L)	64.75 ± 15.12	76.38 ± 27.57	0.081
	Burea nitrogen (mmol/L)	5.57 ± 1.36	6.38 ± 2.33	0.154
	Uric acid (umol/L)	301.45 ± 82.28	341.32 ± 94.65	0.091
	Low-density lipoprotein (mmol/L)	2.23 ± 0.53	2.43 ± 0.79	0.236
	High-density lipoprotein (mmol/L)	1.25 ± 0.36	1.19 ± 0.33	0.500
	Cholesterol (mmol/L)	3.87 ± 0.65	4.07 ± 1.014	0.409
	Triglycerides (mmol/L)	1.35 ± 0.65	1.48 ± 1.02	0.521
	Hemoglobin A1c (%)	6.67 ± 2.03	6.21 ± 1.09	0.265
	Fasting plasma glucose (mmol/L)	5.97 ± 2.70	5.48 ± 2.01	0.474

*IC* intrinsic capacity, *SPPB* short physical performance battery, *MoCA* Montreal cognition assessment, *GDS* Geriatric Depression Scale, *NRS* Nutritional risk screening

*Data were expressed as mean ± standard deviation or n (%)

**Fig. 3:**
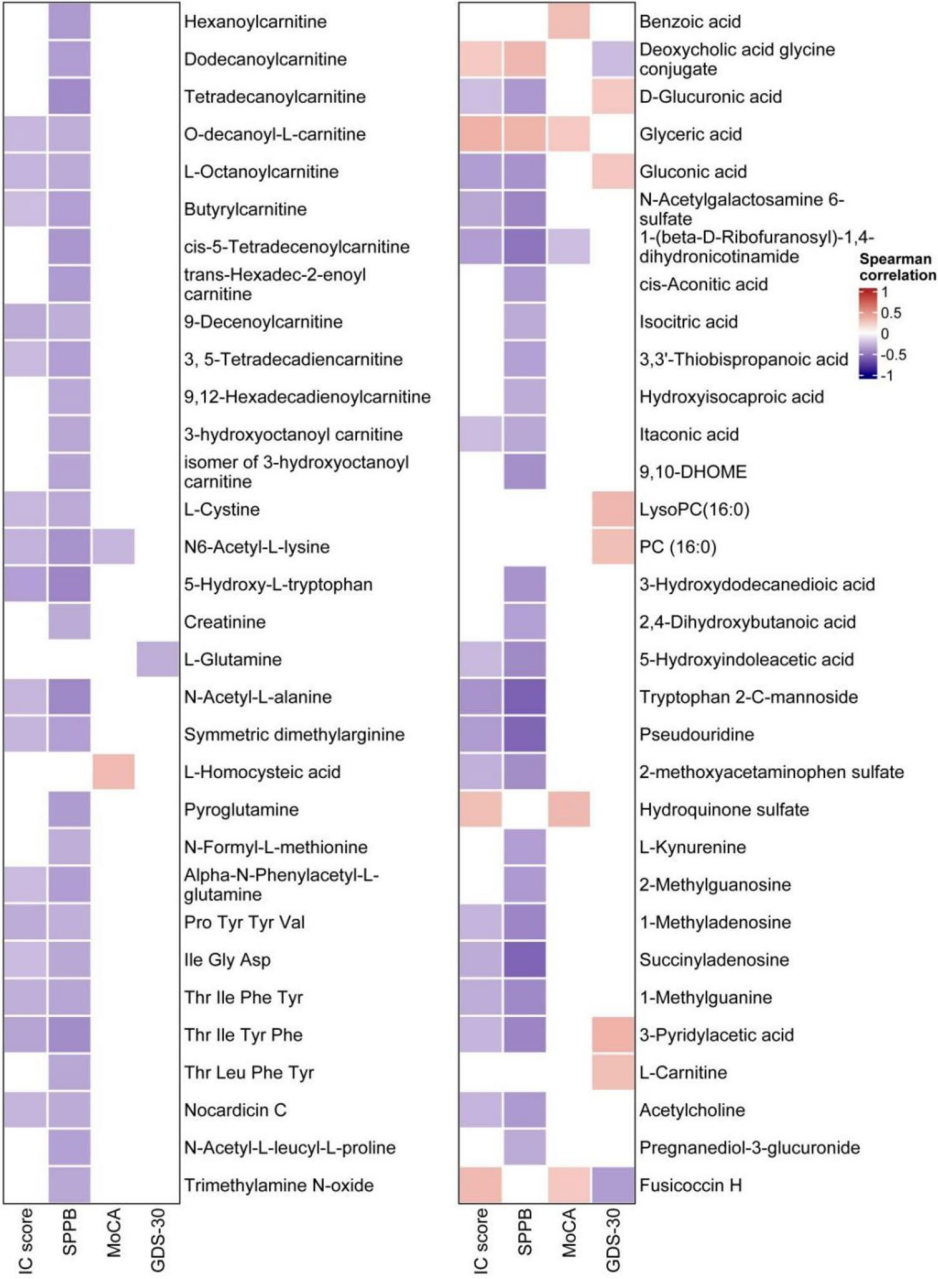
Heat map of Spearman correlation analysis of metabolites associated with intrinsic capacity (IC) score, SPPB, MoCA or GDS-30. The metabolites displayed were those with *p* < 0.05 and absolute value of Spearman rho > 0.3. Negative and positive correlations are shown in blue and red ranging from − 1.0 to 1.0. *IC* intrinsic capacity, *SPPB* short physical performance battery, *MoCA* Montreal cognition assessment, *GDS* geriatric depression scale

A total of 349 compounds in 46 classes were identified from serum in our metabolomic analysis. The OPLS-DA score plots showed that there were significant differences in metabolome characteristics between the decline in IC (and each domain) group and the non-decline group (Fig. 2B–F). Metabolites with FC > 1.5 and *p* < 0.05, or variable important for the projection (VIP) > 1.5 and *p* < 0.05 were considered as potential biomarkers. We identified 24, 54, 14, 6, and 17 differential metabolites between the decline in IC, mobility, cognition, psychology, and sensory groups and their respective non-decline groups (Table S2). Because there were only 2 participants in the vitality decline group, which was incomparable with the control group, we did not analyze it. In the markers of the decline in IC group, 21 were upregulated in serum (including five medium-chain acyl carnitines: 9-decenoylcarnitine, O-decanoyl-l-carnitine, L-octanoylcarnitine, cis-4-decenoylcarnitine and dodecanoylcarnitine; and three long-chain acyl carnitines: cis-5-tetradecenoylcarnitine, 3,5-tetradecadiencarnitine and 9,12-hexadecadienoylcarnitine), and three were down-regulated: glyceric acid, 2-oxoarginine and deoxycholic acid glycine conjugate. Among the above 24 differential compounds of the decline in IC, 15, 7 and 3 were associated with mobility, cognition and sensory declines, but none were associated with psychology impairment. Spearman correlation analysis showed that there were 11 and 33 metabolites positively and negatively correlated with IC score, respectively, all of which were displayed in the heat map (Fig. 3). We performed log10 transformation on the peak intensities of the differential metabolites identified in each group, and then conducted linear regression analysis on them with the score of IC, SPPB, MoCA and GDS-30, adjusting for age and sex. Of the 29 metabolites linearly associated with IC scores, 20, 2 and 8 metabolites were linearly associated with SPPB, MoCA and GDS-30 respectively (Table S3).

Further enrichment analysis identified 5 metabolite sets associated with decline in certain IC domains: carnitine synthesis and glycine and serine metabolism for decline in cognition; purine metabolism and methylhistidine metabolism for decline in psychology; and Bile acid biosynthesis for decline in sensory (Table S4). Pathway analysis identified 13 pathways associated with decline in one or more IC domains (Table S5), of which the IC-associated pathways were D-arginine and D-ornithine metabolism, tryptophan metabolism and pentose phosphate pathway (PPP).

## Discussion

**Fig. 4:**
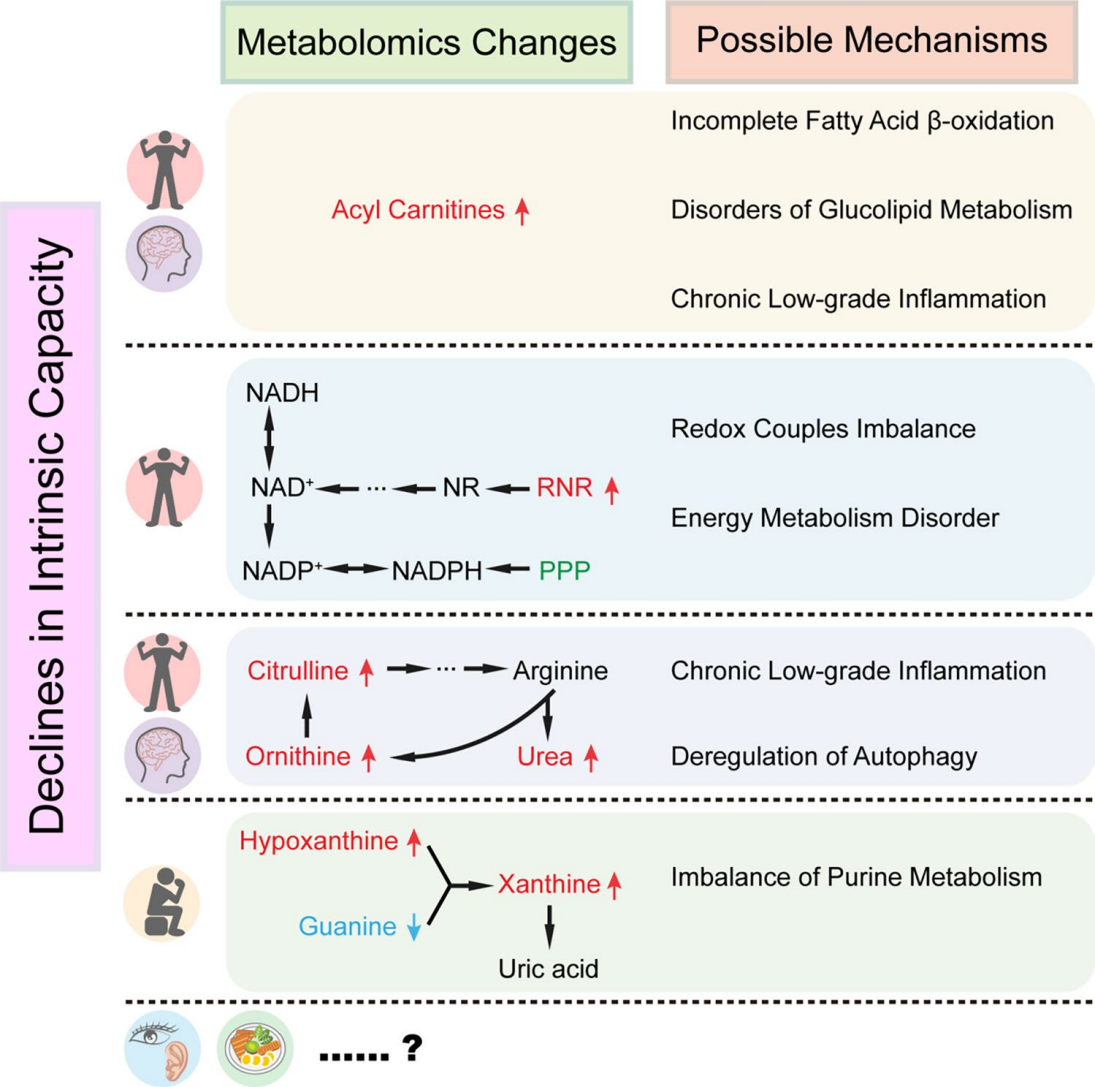
Metabolomics changes and possible mechanism of intrinsic capacity (IC) decline. Up-regulation of certain acyl carnitines is characteristic of IC (especially mobility and cognition) decline; RNR up regulation and PPP disorder are features of IC (especially mobility) decline; urea cycle disturbances are features of IC (especially mobility and cognition) decline; imbalance of purine metabolism is characteristic of decline in psychology; metabolomics signature of sensory and vitality domains was unclear. *IC* intrinsic capacity, *RNR* reduced nicotinamide riboside, *NR* nicotinamide riboside, *NAD + * nicotinamide adenine dinucleotide, *NADH* reduced nicotinamide adenine dinucleotide, *NADP + * nicotinamide adenine dinucleotide phosphate, *NADPH* reduced nicotinamide adenine dinucleotide phosphate, *PPP* pentose phosphate pathway

Our study applied a novel IC score to quantify the level of healthy aging in older adults. By applying untargeted metabolomics, we first explored the cross-sectional associations between serum metabolites and IC scores and each IC domain. We found a set of metabolites that can be considered as candidate biomarkers for IC decline, including 8 acyl carnitines. Dysregulation of PPP and arginine and ornithine metabolism may be underlying mechanisms of IC decline (Fig. 4).

### Acyl carnitines

Acyl carnitines are esters of fatty acids combined with l-carnitine. Its main function is to transport fatty acids to mitochondria for β-oxidation and provide energy for cells. There are 1240 known acyl carnitines in the human body. In our study, eight acyl carnitines (including five medium-chain and three long-chain acyl carnitines) were found to be candidate biomarkers for decline in IC.

Previous studies have found that elevated acyl carnitines suggest incomplete fatty acid β-oxidation, as well as altered carbohydrate and lipid metabolism, and to be associated with a variety of pathological conditions. In previous study, we observed a positive association between the levels of some acyl carnitines and inactivity in older adults [13]. Changes in serum 3,5-tetradecadiencarnitine, l-octanoylcarnitine and cis-5-tetradecenoylcarnitine concentrations were found to be associated with type 2 diabetes and prediabetic status [14], among which, cis-5-tetradecenoylcarnitine is considerde a marker of mitochondrial dysfunction in diabetes [15]. Elevated concentrations of long-chain (C13-C20) and medium-chain (C6-C12) acyl carnitines is also a metabolic hallmark of Alzheimer’s disease [16]. A longitudinal study of over 10 years showed that dodecanoylcarnitine levels were associated with annual reduction in patellar cartilage volume in community-dwelling older adults [17]. Previous studies have shown that tumor necrosis factor receptor-1 and growth differentiation factor-15 related chronic inflammation may be related to the decline in IC [18, 19]. Acyl carnitines may also have pro-inflammatory effects, but the specific targets and involved factors are still unclear [20].

In addition, acylcarnitines have been shown to be predictive of various diseases. High plasma levels of 3,5-tetradecadiencarnitine, L-octanoylcarnitine and dodecanoylcarnitine in patients with type 2 diabetes indicate high risk of coronary heart disease [21]. Plasma levels of long-chain acyl carnitines can be used to predict type 2 diabetes risk [22], and also help diagnose insulin resistance in skeletal and cardiac muscle [23].

Circulating acyl carnitine concentrations can be influenced by factors such as diet and exercise. Weight loss induced by a low calorie diet was accompanied by significant increases in plasma levels of 9-decenoylcarnitine, 3,5-tetradecadiencarnitine, L-octanoylcarnitine, cis-4-decenoylcarnitine, cis-5-tetradecenoylcarnitine, 9,12-hexadecadienoylcarnitine, dodecanoylcarnitine and free fatty acid [24]. However, the PROtein Overfeeding study showed that protein intake was the main factor affecting the changes of acyl carnitines, and various acyl carnitines levels increased in the low-protein diet group [25]. Exercise is known to increase circulating levels of most acyl carnitines [26], and also affects urinary nonanoyl, decanoyl, and ketodecanoylcarnitine concentrations [27].

The importance of acyl carnitines as mitochondrial biomarkers is being gradually recognized [28]. Our results raise the possibility of acyl carnitines as biomarkers of decline in IC for the first time. Whether upregulated acyl carnitine levels in older adults with decline in IC are consequence of incomplete fatty acid β-oxidation and whether low-grade inflammation promoted by acyl carnitine levels is responsible for the decline in IC, need further verification.

### Carbohydrates

The differential metabolites we identified associated with decline in IC included four carbohydrates: 1-(beta-D-ribofuranosyl)-1,4-dihydronicotinamide, gluconic acid and N-acetylgalactosamine 6-sulfate upregulation, and glyceric acid downregulation. Among them, 1-(beta-D-ribofuranosyl)-1,4-dihydronicotinamide is also called reduced nicotinamide riboside (RNR), which is the reduced form of nicotinamide riboside (NR). NR is the precursor of nicotinamide adenine dinucleotide (NAD^+^), which is involved in free radical-mediated production of reactive oxygen species [29]. Downregulation of NAD^+^ can lead to aging-related diseases (such as frailty, sarcopenia, cancer and neurodegeneration), metabolic diseases (such as obesity, fatty liver and type 2 diabetes) and cardiovascular diseases (such as hypertension, arrhythmia and heart failure) [30–32]. Although we did not observe changes in NAD^+^ levels, the upregulation of RNR levels suggested that there may be dysregulation of NAD^+^ in the older adults with decline in IC.

Besides, we also observed the PPP to be associated with the decline in IC. The PPP, an important branch of glucose metabolism, is the main source of reduced nicotinamide adenine dinucleotide phosphate (NADPH) and plays a key role in cellular redox status. NADPH is required for the fatty acids synthesis and the reactive oxygen species (ROS) scavenging, which is important for cellular antioxidant defense. PPP has many biological effects. Animal experiments have found that mice overexpressing glucose-6-phosphate dehydrogenase can promote NADPH production through PPP, thereby reducing age-related hearing loss [33]. PPP is neuroprotective for maintaining brain energy metabolism [34]. Moreover, PPP affects stem cell differentiation and cancer cell growth [35]. In terms of physical function, PPP was associated with frailty phenotype in the elderly [13]. Through genome-wide CRISPR modifier screening approach, some scholars have found that PPP-related genes play an important role in mitochondrial dysfunction [36]. PPP can also control glucose-stimulated insulin secretion by regulating the NADPH/GSH/SENP1 pathway [37]. We speculate that the decline in IC may be related to PPP-related imbalance of intracellular energy metabolism, but it remains to be verified.

### Amino acids

We observed that arginine and ornithine metabolism (with downregulation of 2-oxoarginine) was associated with decline in IC and cognition, and arginine biosynthesis [with upregulation of citrulline (Cit), ornithine (Orn) and urea] was associated with decline in mobility. Arginine (Arg) is a semi-essential amino acid, which is the precursor of nitrogen-containing compounds such as Orn and Cit, which has abundant physiological functions. Arginine and ornithine metabolism are closely related to multiple aging phenotypes [12].

In terms of cognition, animal experiments have found that aging has a significant impact on the arginase metabolic pathway in the brain and the levels of related metabolites such as Arg, Cit and Orn [38, 39]. Cognitive deficits in aged rats were found to be positively correlated with Arg levels [40]. In terms of exercise, the content of Arg-related metabolites in the muscles of mice decreased with the decline of muscle mass and strength [41]. In humans, 45-day Arg supplementation significantly improved athletic performance and VO_2_max in male athletes [42]. Cit was observed to boosts strength, endurance, and walking speed in older adults after 6 weeks of supplementation [43]. Two weeks of exercise with L-Arg supplement showed protective effects on the heart of aged mice, such as reducing myocardial cell damage, apoptosis and remodeling, improving antioxidant capacity, and reducing expression of pro-inflammatory markers like TNF-α, IL-1β, and IL-6 [44]. The inverse correlation of arginine and ornithine metabolism with IC (especially mobility and cognition) is possibly related to the chronic low-grade inflammation induced by Arg and related compounds [45], and the regulation of autophagy related to mammalian target of rapamycin complex 1 (mTORC1) [46].

### Purines

Interestingly, the results of our enrichment analysis and pathway analysis both suggest that purine metabolism (with xanthine and hypoxanthine upregulation, and guanine downregulation) is highly correlated with psychological disorders. The role of purinergic signaling in depression has long received attention. In 1983, a study found that lower levels of hypoxanthine in the cerebrospinal fluid of depressed patients were associated with anger and suicidal tendencies, and lower levels of xanthine were associated with subjective depression [47]. With the application of metabolomics, in the hypothalamus of the mouse model of inflammation-related depression, there was also an imbalance of purine metabolism, which showed that metabolites such as hypoxanthine were significantly down-regulated [48]. In children and adolescents, purine metabolism (with hypoxanthine decrease) is considered as a potential independent diagnostic biomarker in major depressive disorder [49]. The above results are contrary to ours. However, a Finnish study observed perturbations in purine metabolism in patients with major depressive disorder with accumulation of xanthines [50], which supports our findings. A recent perspective suggests that dysfunction of the astrocyte purinergic system may be involved in the pathogenesis of major depressive disorder [51]. Promisingly, dietary supplementation with lactobacillus paracasei *CCFM1229* and *CCFM1228* could modulate purine metabolism by decreasing the xanthine oxidase activity in brain, thereby reducing anxiety and depression phenotypes in a mouse model of depression [52]. Our metabolomic results suggest that purine metabolism also plays an important role in depression among older adults. In the future, it appears as a promising strategy to intervene purine metabolism through dietary supplements or microbial supplements to regulate the psychological state of older adults.

### Strengths and limitations

In our study, IC score was applied to quantify IC for clarifying the linear relationship between metabolites and the degree of IC, and to avoid the information loss caused by data analysis when IC is simply classified into two categories. Besides, we explored the relationship between IC (and each domain) and metabolome, and compared the similarities and differences between IC and each domain in terms of metabolomics characteristics. Furthermore, we analyzed a large number of compounds with the untargeted metabolomics platform, which made our analysis more comprehensive and avoided overlooking some potential associations between IC and metabolites.

This study also had several important limitations. First, cross-sectional study designs cannot establish causal relationships between changes in metabolites and pathways and clinical phenotypes, or distinguish the decline trajectory of intrinsic capacity. Second, the limited sample size led to insufficient extrapolation and replicability of the results. Third, in the assessment of sensory domain, we adopted self-reported impairments instead of optometry or audiometry, which may have some subjectivity. Besides, as comorbidities and polypharmacy are common in older adults, it is difficult to avoid drug interference with our results. In particular, psychotropic drugs such as benzodiazepines may affect cognitive performance. Although the participants in this study had no long-term history of psychotropic drug use, however, the effects of other drugs on IC and metabolism are unknown. In metabolomics analysis, we only collected blood samples at a specific time, so we might have ignored metabolites changes that depend on the circadian clock. In addition, metabolites are greatly affected by diet and exercise, but we lack the quantification and comparison of these elements. Although we tried to minimize the influence of diet by collecting blood samples upon fasting for more than 8 h, it inevitably affects the interpretation of some metabolites. Finally, we only analyzed those metabolites that could be detected and identified by our metabolomics platform. We obtained and compared the peak intensities of metabolites, which lacked comparability with their normal values of serum concentration.

## Conclusion

Multi-dimensionality is the essential feature of IC, which also determines the complexity of its mechanism and metabolomics characteristics. We quantified IC levels with the IC score and applied metabolomics approach to identify multiple metabolites associated with IC. We propose a panel of biomarkers of decline in IC, including changes in various acyl carnitines, carbohydrates, amino acids and other compounds. Dysregulation of acyl carnitines, PPP, and arginine and ornithine metabolism are the possible mechanisms involved in IC decline. Our finding highlights that purine metabolism may be an important pathway of psychological disorders. Furthermore, in the future, we may be able to build animal models with specific pathway changes or conduct interventional research based on existing metabolomics results. Longitudinal studies should also be carried out in different populations, so as to collect ample evidence for enabling us to further design clinical trials for optimizing IC in older adults.

### Supplementary Information

Below is the link to the electronic supplementary material.

Supplementary file1 (DOCX 179 KB)

## Data Availability

The data is not available online to protect participant privacy. If required, they can be obtained by contacting the corresponding author.
