# Supplementary material for: Dysregulation of acyl carnitines, pentose phosphate pathway and arginine and ornithine metabolism are associated with decline in intrinsic capacity in Chinese older adults
Source: Aging Clin Exp Res. 2024 Feb 12;36(1):36. doi: 10.1007/s40520-023-02654-x (PMC10861606; doi:10.1007/s40520-023-02654-x)
Supplement: Supplementary file 1 — Supplementary file1 (DOCX 179 KB) [file 40520_2023_2654_MOESM1_ESM.docx]

**Figure S1** Flow chart of the study.

**
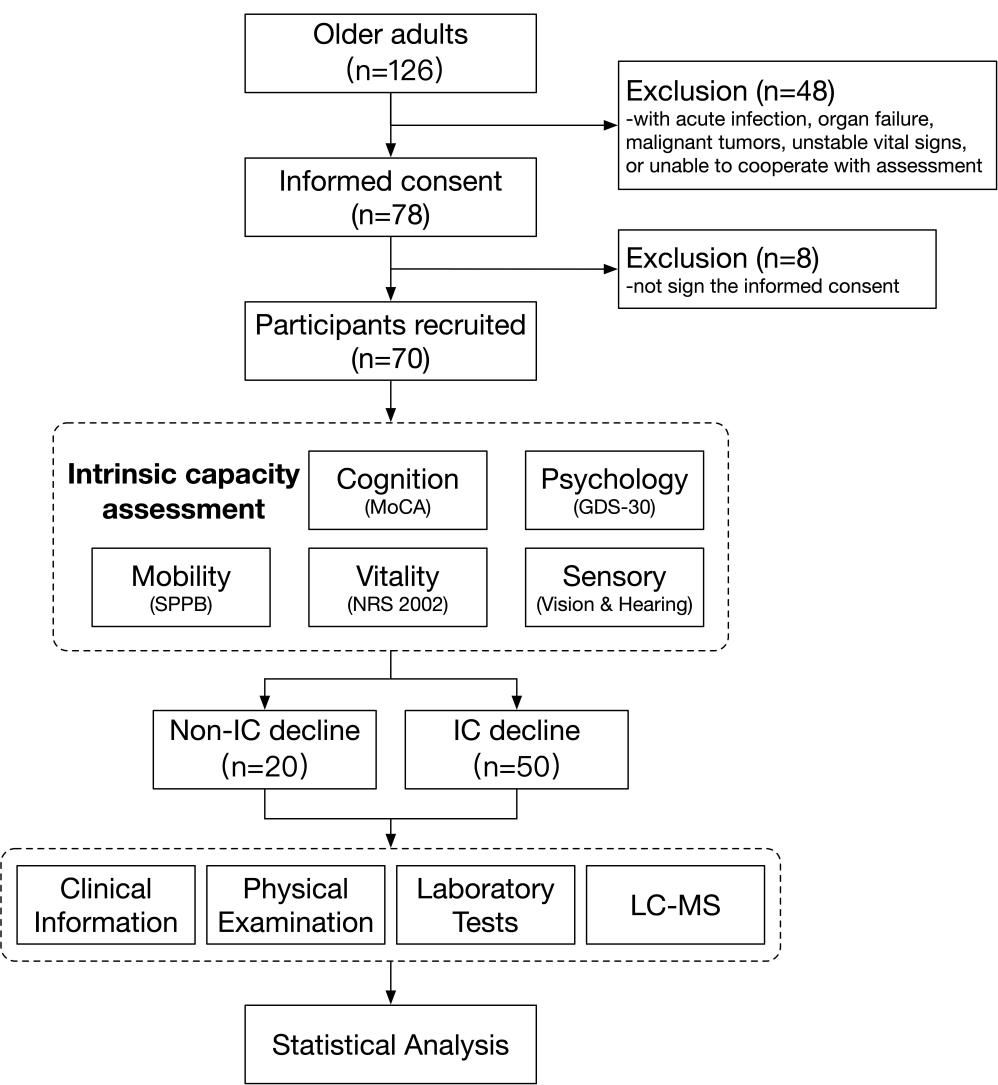
**

**Table S1.** Evaluation tools and cut-off points for each dimension of intrinsic capacity (IC).

| **Domain of IC** | **Weighted scores of each domain** |
| --- | --- |
| Mobility | 0: SPPB 0-3 |
|  | 1: SPPB 4-9 |
|  | 2: SPPB 10-12 |
| Cognition | 0: MoCA ≤21 |
|  | 1: MoCA 22-25 |
|  | 2: MoCA ≥26 |
| Psychology | 0: GDS 21-30 |
|  | 1: GDS 11-20 |
|  | 2: GDS 0-10 |
| Sensory | 0: Both hearing and visual impairment |
|  | 1: Either hearing or visual impairment |
|  | 2: No hearing or visual impairment |
| Vitality | 0: NRS-2002 ≥5 |
|  | 1: NRS-2002 3-4 |
|  | 2: NRS-2002 0-2 |

Abbreviations: IC, intrinsic capacity; SPPB, short physical performance battery; MoCA, Montreal cognition assessment; GDS, geriatric depression scale; NRS, nutritional risk screening.

*MoCA scores were adjusted for age. If the years of education is less than 13 years, add 1 to the MoCA score.

**Table S2.** Differential metabolites associated with decline in intrinsic capacity (IC) and each domain.

| **Domain of IC** | **Differential metabolites** | **Database ID** | **Class** | **FC** | **p-value** | **VIP** |
| --- | --- | --- | --- | --- | --- | --- |
| IC | 9-Decenoylcarnitine | HMDB0013205 | Acyl carnitines | 1.73 | 0.0085 | 2.00 |
|  | 3, 5-Tetradecadiencarnitine | HMDB0013331 | Acyl carnitines | 1.56 | 0.0133 | 2.33 |
|  | 9,12-Hexadecadienoylcarnitine | HMDB0013334 | Acyl carnitines | 1.46 | 0.0189 | 2.16 |
|  | O-decanoyl-L-carnitine | HMDB0062631 | Acyl carnitines | 1.46 | 0.0128 | 2.08 |
|  | cis-5-Tetradecenoylcarnitine | HMDB0002014 | Acyl carnitines | 1.45 | 0.0304 | 2.22 |
|  | L-Octanoylcarnitine | HMDB0000791 | Acyl carnitines | 1.39 | 0.0137 | 1.87 |
|  | cis-4-Decenoylcarnitine | HMDB0240585 | Acyl carnitines | 1.38 | 0.0249 | 2.04 |
|  | Dodecanoylcarnitine | HMDB0002250 | Acyl carnitines | 1.25 | 0.0412 | 1.98 |
|  | Thr Ile Tyr Phe | METLIN 234663 | Amino acids | 1.51 | 0.0446 | 1.76 |
|  | Pro Tyr Tyr Val | METLIN 207476 | Amino acids | 1.48 | 0.0358 | 1.57 |
|  | Thr Ile Phe Tyr | METLIN 234378 | Amino acids | 1.45 | 0.0267 | 1.96 |
|  | 5-Hydroxy-L-tryptophan | HMDB0000472 | Amino acids | 1.21 | 0.0346 | 1.74 |
|  | 1-(beta-D-Ribofuranosyl)-1,4-dihydronicotinamide | HMDB0011648 | Carbohydrates | 1.27 | 0.0159 | 1.78 |
|  | N-Acetylgalactosamine 6-sulfate | HMDB0000841 | Carbohydrates | 1.24 | 0.0276 | 1.58 |
|  | Gluconic acid | HMDB0000625 | Carbohydrates | 1.21 | 0.0346 | 1.54 |
|  | 3-Oxododecanoic acid | HMDB0010727 | Fatty acids | 1.34 | 0.0237 | 1.53 |
|  | Tryptophan 2-C-mannoside | HMDB0240296 | Indolyl carboxylic acids | 1.30 | 0.0041 | 1.84 |
|  | Pseudouridine | HMDB0000767 | Nucleosides | 1.26 | 0.0210 | 1.72 |
|  | Indoxyl sulfate | HMDB0000682 | Organic sulfuric acids | 1.90 | 0.0106 | 1.35 |
|  | Hydroquinone sulfate | HMDB0240263 | Organic sulfuric acids | 1.76 | 0.0186 | 1.53 |
|  | 1-Methylguanine | HMDB0003282 | Purines | 1.18 | 0.0165 | 2.12 |
|  | 2-Oxoarginine | HMDB0004225 | Keto acids | 0.85 | 0.0304 | 1.57 |
|  | Glyceric acid | HMDB0000139 | Carbohydrates | 0.77 | 0.0031 | 2.22 |
|  | Deoxycholic acid glycine conjugate | HMDB0000631 | Bile acids | 0.59 | 0.0040 | 1.22 |
| Mobility | p-Hydroxyphenylacetic acid | HMDB0000020 | Phenols | 3.42 | 0.0454 | 0.35 |
|  | Chenodeoxycholic acid 3-sulfate | HMDB0002586 | Bile acids | 3.26 | 0.0106 | 1.31 |
|  | Cholic acid | HMDB0000619 | Bile acids | 2.15 | 0.0365 | 1.23 |
|  | Isomer of glycochenodeoxycholic acid 3-glucuronide |  | Steroidal glycosides | 1.83 | 0.0068 | 1.63 |
|  | Glycochenodeoxycholic acid 3-glucuronide | HMDB0002579 | Steroidal glycosides | 1.83 | 0.0068 | 1.63 |
|  | Alpha-N-Phenylacetyl-L-glutamine | HMDB0006344 | Amino acids | 1.69 | 0.0217 | 1.85 |
|  | 2,4-Dihydroxybutanoic acid | HMDB0000360 | Hydroxy acids | 1.62 | 0.0045 | 1.08 |
|  | Thr Ile Tyr Phe | METLIN 234663 | Amino acids | 1.60 | 0.0059 | 1.88 |
|  | 3-Hydroxydodecanedioic acid | HMDB0000413 | Hydroxy acids | 1.60 | 0.0009 | 1.92 |
|  | cis-5-Tetradecenoylcarnitine | HMDB0002014 | Acyl carnitines | 1.56 | 0.0036 | 2.32 |
|  | 3, 5-Tetradecadiencarnitine | HMDB0013331 | Acyl carnitines | 1.52 | 0.0085 | 2.11 |
|  | Succinyladenosine | HMDB0000912 | Purine nucleosides | 1.48 | ＜0.0001 | 2.24 |
|  | Pseudouridine | HMDB0000767 | Nucleosides | 1.42 | ＜0.0001 | 2.23 |
|  | Indolelactic acid | HMDB0000671 | Indolyl carboxylic acids | 1.42 | 0.0084 | 1.67 |
|  | 9,12-Hexadecadienoylcarnitine | HMDB0013334 | Acyl carnitines | 1.42 | 0.0154 | 2.02 |
|  | Thr Ile Phe Tyr | METLIN 234378 | Amino acids | 1.42 | 0.0245 | 1.63 |
|  | Tryptophan 2-C-mannoside | HMDB0240296 | Indolyl carboxylic acids | 1.42 | ＜0.0001 | 2.21 |
|  | Pyroglutamine | HMDB0062558 | Amino acids | 1.40 | 0.0022 | 1.91 |
|  | 3-hydroxyoctanoyl carnitine | HMDB0061634 | Acyl carnitines | 1.40 | 0.0382 | 1.65 |
|  | isomer of 3-hydroxyoctanoyl carnitine |  | Acyl carnitines | 1.38 | 0.0139 | 1.77 |
|  | 1-(beta-D-Ribofuranosyl)-1,4-dihydronicotinamide | HMDB0011648 | Carbohydrates | 1.38 | 0.0001 | 2.05 |
|  | Pregnanediol-3-glucuronide | HMDB0010318 | Steroidal glycosides | 1.37 | 0.0458 | 1.64 |
|  | 9,10-DHOME | HMDB0004704 | Fatty acids | 1.37 | 0.0008 | 1.99 |
|  | 5-Hydroxyindoleacetic acid | HMDB0000763 | Indoles | 1.36 | 0.0003 | 1.75 |
|  | O-decanoyl-L-carnitine | HMDB0062631 | Acyl carnitines | 1.33 | 0.0319 | 1.57 |
|  | trans-Hexadec-2-enoyl carnitine | HMDB0006317 | Acyl carnitines | 1.33 | 0.0038 | 2.54 |
|  | 2-methoxyacetaminophen sulfate | HMDB0062550 | Organic sulfuric acids | 1.32 | 0.0024 | 1.78 |
|  | N-Acetylgalactosamine 6-sulfate | HMDB0000841 | Carbohydrates | 1.32 | 0.0018 | 1.88 |
|  | 3-Pyridylacetic acid | HMDB0001538 | Pyridines | 1.32 | 0.0026 | 1.59 |
|  | Dodecanoylcarnitine | HMDB0002250 | Acyl carnitines | 1.31 | 0.0082 | 1.94 |
|  | Itaconic acid | HMDB0002092 | Fatty acids | 1.29 | 0.0126 | 1.60 |
|  | 5-Hydroxy-L-tryptophan | HMDB0000472 | Amino acids | 1.27 | 0.0012 | 1.96 |
|  | N-Formyl-L-methionine | HMDB0001015 | Amino acids | 1.27 | 0.0250 | 1.51 |
|  | D-Glucuronic acid | HMDB0000127 | Carbohydrates | 1.26 | 0.0150 | 1.63 |
|  | L-Kynurenine | HMDB0000684 | Organooxygen | 1.25 | 0.0059 | 1.68 |
|  | N-Acetyl-L-alanine | HMDB0000766 | Amino acids | 1.24 | 0.0012 | 2.08 |
|  | Tetradecanoylcarnitine | HMDB0005066 | Acyl carnitines | 1.24 | 0.0006 | 2.45 |
|  | 1-Methylguanine | HMDB0003282 | Purines | 1.23 | 0.0011 | 2.31 |
|  | 4-Guanidinobutanoic acid | HMDB0003464 | Amino acids | 1.22 | 0.0498 | 1.59 |
|  | cis-Aconitic acid | HMDB0000072 | Carboxylic acids | 1.21 | 0.0047 | 1.84 |
|  | Creatinine | HMDB0000562 | Amino acids | 1.20 | 0.0203 | 1.55 |
|  | 1-Methyladenosine | HMDB0003331 | Purine nucleosides | 1.20 | 0.0049 | 1.67 |
|  | Acetylcholine | HMDB0000895 | Quaternary ammonium salts | 1.19 | 0.0015 | 1.96 |
|  | Hexanoylcarnitine | HMDB0000756 | Acyl carnitines | 1.18 | 0.0108 | 1.56 |
|  | L-Palmitoylcarnitine | HMDB0000222 | Acyl carnitines | 1.18 | 0.0387 | 1.79 |
|  | N6-Acetyl-L-lysine | HMDB0000206 | Amino acids | 1.17 | 0.0009 | 1.67 |
|  | Citric acid | HMDB0000094 | Carboxylic acids | 1.17 | 0.0350 | 1.71 |
|  | Symmetric dimethylarginine | HMDB0001539 | Amino acids | 1.15 | 0.0036 | 1.74 |
|  | L-Lysine | HMDB0000182 | Amino acids | 0.92 | 0.0260 | 1.56 |
|  | L-2-Aminoadipic acid | PubChem 92136 | Amino acids | 0.83 | 0.0242 | 1.95 |
|  | Glyceric acid | HMDB0000139 | Carbohydrates | 0.77 | 0.0018 | 1.81 |
|  | 3-(2,3,4-trimethoxyphenyl)propanoic acid | HMDB0142074 | Phenylpropanoic acids | 0.66 | 0.0416 | 1.44 |
|  | Phenylgalactoside | METLIN 65793 | Glycosides | 0.64 | 0.0116 | 1.77 |
|  | Deoxycholic acid glycine conjugate | HMDB0000631 | Bile acids | 0.58 | 0.0085 | 0.91 |
| Cognition | Fusicoccin H | METLIN 67422 | Steroidal glycosides | 2.20 | 0.0113 | 0.82 |
|  | Testosterone sulfate | HMDB0002833 | Sulfated steroids | 1.52 | 0.0171 | 1.82 |
|  | O-decanoyl-L-carnitine | HMDB0062631 | Acyl carnitines | 1.39 | 0.0366 | 2.23 |
|  | Thr Ile Phe Tyr | METLIN 234378 | Amino acids | 1.39 | 0.0499 | 1.78 |
|  | L-Octanoylcarnitine | HMDB0000791 | Acyl carnitines | 1.34 | 0.0324 | 2.07 |
|  | Glycine | HMDB0000123 | Amino acids | 1.19 | 0.0344 | 2.69 |
|  | L-Serine | HMDB0000187 | Amino acids | 1.15 | 0.0480 | 2.19 |
|  | L-Homocysteic acid | HMDB0002205 | Amino acids | 0.87 | 0.0499 | 1.59 |
|  | 2-Oxoarginine | HMDB0004225 | Keto acids | 0.83 | 0.0101 | 2.46 |
|  | Glyceric acid | HMDB0000139 | Carbohydrates | 0.79 | 0.0026 | 2.42 |
|  | 4-Hydroxyphenylpyruvic acid | HMDB0000707 | Benzene | 0.78 | 0.0426 | 2.55 |
|  | N-Acetylmuramate | HMDB0060493 | Carbohydrates | 0.73 | 0.0329 | 2.23 |
|  | 9-Decenoylcarnitine | HMDB0013205 | Acyl carnitines | 0.61 | 0.0133 | 2.33 |
|  | Hydroquinone sulfate | HMDB0240263 | Organic sulfuric acids | 0.47 | 0.0195 | 0.35 |
| Psychology | Fusicoccin H | METLIN 67422 | Steroidal glycosides | 3.27 | 0.0108 | 0.04 |
|  | Muramic acid | HMDB0003254 | Carbohydrates | 1.45 | 0.0389 | 1.77 |
|  | LysoPE(18:2) | HMDB0011507 | Glycerophosphoethanolamines | 1.43 | 0.0253 | 2.23 |
|  | sn2 LysoPE(18:2) |  | Glycerophosphoethanolamines | 1.33 | 0.0147 | 2.26 |
|  | L-Carnitine | HMDB0000062 | Quaternary ammonium salts | 1.18 | 0.0032 | 2.17 |
|  | L-Homoserine | HMDB0000719 | Amino acids | 1.13 | 0.0248 | 1.55 |
| Sensory | Tyrosyl-Glutamate | HMDB0029104 | Amino acids | 1.78 | 0.0305 | 2.23 |
|  | Thr Ile Tyr Phe | METLIN 234663 | Amino acids | 1.60 | 0.0204 | 1.87 |
|  | 2,4-dihydroxy-3-methoxybenzoic acid | HMDB0125542 | Benzoic acids | 1.46 | 0.0057 | 1.84 |
|  | 13-HODE | HMDB0004667 | Fatty Acyls | 1.35 | 0.0470 | 1.61 |
|  | 2,5-dihydroxy-4-methoxybenzoic acid | HMDB0130482 | Benzoic acids | 1.31 | 0.0249 | 1.55 |
|  | 2-Methoxyhydroquinone | METLIN 263518 | Phenols | 1.29 | 0.0293 | 1.61 |
|  | 1-Methylguanine | HMDB0003282 | Purines | 1.20 | 0.0085 | 2.12 |
|  | Gamma-Glutamyl Glutamine | HMDB0028833 | Amino acids | 1.18 | 0.0344 | 2.24 |
|  | Phosphorylcholine | HMDB0001565 | Quaternary ammonium salts | 1.16 | 0.0044 | 1.82 |
|  | N-gamma-L-Glutamyl-L-methionine | HMDB0034367 | Amino acids | 1.15 | 0.0160 | 1.75 |
|  | L-Leucine | HMDB0000687 | Amino acids | 0.92 | 0.0203 | 2.24 |
|  | N-Acetylmuramate | HMDB0060493 | Carbohydrates | 0.68 | 0.0045 | 2.29 |
|  | sn3 LysoPC(20:5) |  | Glycerophosphocholines | 0.59 | 0.0136 | 1.77 |
|  | LysoPC(20:5) | HMDB0010397 | Glycerophosphocholines | 0.59 | 0.0499 | 1.74 |
|  | Taurodeoxycholic acid | HMDB0000896 | Bile acids | 0.46 | 0.0035 | 2.25 |
|  | Nornicotine | HMDB0001126 | Pyridines | 0.31 | 0.0131 | 2.08 |
|  | Deoxycholic acid glycine conjugate | HMDB0000631 | Bile acids | 0.27 | 0.0131 | 2.81 |

**Table S3.** Linear regression between metabolites and intrinsic capacity (IC).

| **IC score** | | | |  | **SPPB** | | | |  | **MoCA** | | | |  | **GDS-30** | | | |
| --- | --- | --- | --- | --- | --- | --- | --- | --- | --- | --- | --- | --- | --- | --- | --- | --- | --- | --- |
| **Metabolite** | **Estimate** | **P value** | **R^2^** |  | **Metabolite** | **Estimate** | **P value** | **R^2^** |  | **Metabolite** | **Estimate** | **P value** | **R^2^** |  | **Metabolite** | **Estimate** | **P value** | **R^2^** |
| Glyceric acid | 4.399 | 0.004 | 0.218 |  | 3-Pyridylacetic acid | -10.664 | <0.001 | 0.410 |  | N6,N6,N6-Trimethyl-L-lysine | -13.656 | 0.016 | 0.139 |  | 1-Methylguanosine | -7.548 | <0.001 | 0.191 |
| Fusicoccin H | 1.269 | 0.004 | 0.216 |  | Tryptophan 2-C-mannoside | -13.240 | <0.001 | 0.401 |  | L-Homocysteic acid | 9.503 | 0.027 | 0.127 |  | L-Carnitine | 22.664 | 0.002 | 0.146 |
| Tryptophan 2-C-mannoside | -5.136 | 0.005 | 0.211 |  | Pseudouridine | -12.740 | <0.001 | 0.393 |  | Indole-3-carboxylic acid | -12.165 | 0.033 | 0.122 |  | Stearoylcarnitine | 11.774 | 0.003 | 0.132 |
| 1-(beta-D-Ribofuranosyl)-1,4-dihydronicotinamide | -5.070 | 0.005 | 0.211 |  | 1-(beta-D-Ribofuranosyl)-1,4-dihydronicotinamide | -12.286 | <0.001 | 0.378 |  | L-Alanine | -15.537 | 0.035 | 0.121 |  | Arachidyl carnitine | 10.391 | 0.003 | 0.131 |
| 2-methoxyacetaminophen sulfate | -4.825 | 0.008 | 0.201 |  | N-Acetylgalactosamine 6-sulfate | -10.716 | <0.001 | 0.361 |  | Glyceric acid | 8.848 | 0.036 | 0.120 |  | 12a-Hydroxy-3-oxocholadienic acid | -6.596 | 0.004 | 0.129 |
| 3-Pyridylacetic acid | -3.757 | 0.010 | 0.198 |  | 2,4-Dihydroxybutanoic acid | -7.169 | <0.001 | 0.354 |  | Hydroquinone sulfate | 2.737 | 0.038 | 0.120 |  | Fusicoccin H | -2.938 | 0.004 | 0.128 |
| N-Acetylgalactosamine 6-sulfate | -4.250 | 0.012 | 0.194 |  | 2-methoxyacetaminophen sulfate | -11.278 | <0.001 | 0.351 |  | Adenosine | -5.892 | 0.038 | 0.119 |  | Arg Ser Phe | 7.287 | 0.007 | 0.115 |
| sn2 LysoPC (22:4) | -3.655 | 0.013 | 0.192 |  | N-Acetyl-L-alanine | -13.661 | <0.001 | 0.344 |  | 2-Ketobutyric acid | 4.593 | 0.040 | 0.118 |  | sn2 LysoPE(18:2) | 10.778 | 0.007 | 0.112 |
| Arg Ser Phe | -2.815 | 0.017 | 0.187 |  | Succinyladenosine | -10.395 | <0.001 | 0.341 |  | Alpha-N-Phenylacetyl-L-glutamine | -3.624 | 0.041 | 0.118 |  | 3-Pyridylacetic acid | 8.680 | 0.009 | 0.106 |
| Thr Ile Tyr Phe | -1.748 | 0.017 | 0.186 |  | 1-Methylguanine | -14.185 | 0.001 | 0.331 |  | 4-Pyridoxic acid | 4.970 | 0.048 | 0.114 |  | Leucylalanine | -5.363 | 0.009 | 0.106 |
| Gluconic acid | -4.018 | 0.017 | 0.186 |  | Gluconic acid | -8.932 | 0.002 | 0.314 |  | Betaine | 12.085 | 0.050 | 0.113 |  | Cortisol | 7.733 | 0.010 | 0.106 |
| 5-Hydroxy-L-tryptophan | -4.450 | 0.018 | 0.185 |  | Glyceric acid | 7.919 | 0.003 | 0.308 |  |  |  |  |  |  | Glycochenodeoxycholic acid 3-glucuronide | -1.405 | 0.010 | 0.106 |
| Pseudouridine | -4.288 | 0.019 | 0.184 |  | Butyrylcarnitine | -7.633 | 0.003 | 0.305 |  |  |  |  |  |  | Isomer of glycochenodeoxycholic acid 3-glucuronide | -1.405 | 0.010 | 0.106 |
| Cortisol | -3.057 | 0.019 | 0.183 |  | N6-Acetyl-L-lysine | -14.423 | 0.004 | 0.303 |  |  |  |  |  |  | Linoelaidyl carnitine | 7.651 | 0.010 | 0.105 |
| N6,N6,N6-Trimethyl-L-lysine | -4.623 | 0.027 | 0.176 |  | Thr Ile Tyr Phe | -3.645 | 0.004 | 0.302 |  |  |  |  |  |  | LysoPC(16:0) | 12.930 | 0.010 | 0.105 |
| Symmetric dimethylarginine | -7.036 | 0.028 | 0.176 |  | N-Acetylvaline | -8.347 | 0.004 | 0.301 |  |  |  |  |  |  | PC (16:0) | 8.038 | 0.011 | 0.103 |
| sn3 LysoPC(20:5) | 2.161 | 0.030 | 0.174 |  | D-Glucuronic acid | -7.182 | 0.004 | 0.300 |  |  |  |  |  |  | L-Serine | 12.861 | 0.012 | 0.100 |
| Alpha-Ketooctanoic acid | 2.476 | 0.032 | 0.172 |  | Ile Gly Asp | -6.874 | 0.004 | 0.300 |  |  |  |  |  |  | 7-Hydroxyterpineol 8-glucoside | 3.500 | 0.014 | 0.096 |
| Docosahexaenoic acid | 2.432 | 0.034 | 0.171 |  | 1-Methyladenosine | -10.161 | 0.005 | 0.298 |  |  |  |  |  |  | Oleoylcarnitine | 7.938 | 0.016 | 0.094 |
| Theobromine | 0.951 | 0.037 | 0.170 |  | Urea | -9.657 | 0.005 | 0.296 |  |  |  |  |  |  | D-Glucuronic acid | 8.016 | 0.016 | 0.093 |
| Ile Gly Asp | -2.933 | 0.037 | 0.170 |  | 3,3'-Thiobispropanoic acid | -7.150 | 0.006 | 0.295 |  |  |  |  |  |  | Glycerophosphocholine | 9.276 | 0.018 | 0.091 |
| Thr Ile Phe Tyr | -1.755 | 0.037 | 0.169 |  | N6,N6,N6-Trimethyl-L-lysine | -9.619 | 0.008 | 0.289 |  |  |  |  |  |  | LysoPE(18:2) | 7.084 | 0.018 | 0.091 |
| Pro Tyr Tyr Val | -1.669 | 0.037 | 0.169 |  | 5-Hydroxy-L-tryptophan | -8.604 | 0.008 | 0.287 |  |  |  |  |  |  | LysoPC (22:4) | 5.850 | 0.019 | 0.090 |
| 1-Methylguanosine | 1.882 | 0.044 | 0.166 |  | Symmetric dimethylarginine | -14.491 | 0.009 | 0.286 |  |  |  |  |  |  | Alanylleucineine | -3.973 | 0.019 | 0.089 |
| N6-Acetyl-L-lysine | -5.818 | 0.045 | 0.165 |  | Isobutyrylcarnitine | -4.552 | 0.012 | 0.280 |  |  |  |  |  |  | L-Palmitoylcarnitine | 8.215 | 0.021 | 0.087 |
| N-Acetylmuramate | 1.684 | 0.046 | 0.165 |  | sn2 LysoPC (22:4) | -6.442 | 0.012 | 0.280 |  |  |  |  |  |  | 3-carboxy-4-methyl-5-pentyl-2-furanpropanoic acid | -5.457 | 0.023 | 0.085 |
| Butyrylcarnitine | -3.022 | 0.047 | 0.164 |  | Deoxycholic acid glycine conjugate | 0.920 | 0.013 | 0.279 |  |  |  |  |  |  | L-Proline | -14.065 | 0.023 | 0.084 |
| N-Acetyl-L-alanine | -4.564 | 0.047 | 0.164 |  | Tetradecanoylcarnitine | -9.298 | 0.013 | 0.278 |  |  |  |  |  |  | Isobutyrylcarnitine | 5.380 | 0.024 | 0.083 |
| Ser Phe Val Phe | 2.117 | 0.048 | 0.164 |  | Pro Tyr Tyr Val | -3.424 | 0.014 | 0.277 |  |  |  |  |  |  | sn2 LysoPC (22:4) | 7.606 | 0.025 | 0.082 |
|  |  |  |  |  | Indolelactic acid | -5.866 | 0.014 | 0.277 |  |  |  |  |  |  | Arabinonic acid | 7.123 | 0.026 | 0.082 |
|  |  |  |  |  | isomer of Fusicoccin H | 1.649 | 0.015 | 0.276 |  |  |  |  |  |  | L-Valine | 9.271 | 0.033 | 0.076 |
|  |  |  |  |  | Isovalerylcarnitine | -6.130 | 0.018 | 0.272 |  |  |  |  |  |  | Allochenodeoxycholic acid | -2.699 | 0.035 | 0.074 |
|  |  |  |  |  | Thr Ile Phe Tyr | -3.432 | 0.018 | 0.271 |  |  |  |  |  |  | sn2 LysoPE(16:0) | 6.889 | 0.035 | 0.074 |
|  |  |  |  |  | Hexanoylcarnitine | -6.726 | 0.019 | 0.271 |  |  |  |  |  |  | Butyrylcarnitine | 7.261 | 0.037 | 0.073 |
|  |  |  |  |  | 3-Hydroxydodecanedioic acid | -4.086 | 0.021 | 0.269 |  |  |  |  |  |  | 13-HODE | 3.609 | 0.039 | 0.072 |
|  |  |  |  |  | Etiocholanolone glucuronide | -3.340 | 0.023 | 0.267 |  |  |  |  |  |  | L-Methionine | -4.781 | 0.039 | 0.072 |
|  |  |  |  |  | Acetylcholine | -9.986 | 0.026 | 0.265 |  |  |  |  |  |  | sn2 LysoPC(16:0) | 7.397 | 0.040 | 0.071 |
|  |  |  |  |  | Thr Tyr Phe Ile | -2.595 | 0.027 | 0.264 |  |  |  |  |  |  | LysoPC(18:1) | 7.477 | 0.040 | 0.071 |
|  |  |  |  |  | L-gamma-glutamyl-L-isoleucine | -5.345 | 0.027 | 0.264 |  |  |  |  |  |  | Gluconic acid | 7.817 | 0.045 | 0.068 |
|  |  |  |  |  | Thr Leu Phe Tyr | -3.125 | 0.028 | 0.263 |  |  |  |  |  |  | Prolylhydroxyproline | -5.175 | 0.049 | 0.066 |
|  |  |  |  |  | 3, 5-Tetradecadiencarnitine | -3.503 | 0.030 | 0.262 |  |  |  |  |  |  | LysoPC(16:1) | 5.825 | 0.049 | 0.066 |
|  |  |  |  |  | Alpha-N-Phenylacetyl-L-glutamine | -2.408 | 0.033 | 0.260 |  |  |  |  |  |  |  |  |  |  |
|  |  |  |  |  | N-Acetyl-L-leucyl-L-proline | -5.954 | 0.033 | 0.260 |  |  |  |  |  |  |  |  |  |  |
|  |  |  |  |  | 9,10-DHOME | -4.933 | 0.038 | 0.258 |  |  |  |  |  |  |  |  |  |  |
|  |  |  |  |  | Murocholic acid | 2.174 | 0.040 | 0.256 |  |  |  |  |  |  |  |  |  |  |
|  |  |  |  |  | Creatinine | -7.506 | 0.041 | 0.256 |  |  |  |  |  |  |  |  |  |  |
|  |  |  |  |  | 9-Decenoylcarnitine | -3.005 | 0.043 | 0.255 |  |  |  |  |  |  |  |  |  |  |
|  |  |  |  |  | cis-5-Tetradecenoylcarnitine | -3.333 | 0.043 | 0.255 |  |  |  |  |  |  |  |  |  |  |
|  |  |  |  |  | Cortisol | -4.576 | 0.046 | 0.254 |  |  |  |  |  |  |  |  |  |  |
|  |  |  |  |  | (25S)-11alpha,20,26-trihydroxyecdysone | -3.513 | 0.046 | 0.254 |  |  |  |  |  |  |  |  |  |  |

Abbreviations: IC, intrinsic capacity; SPPB, short physical performance battery; MoCA, Montreal cognition assessment; GDS, Geriatric Depression Scale.

*Regression coefficient obtained by linear regression between intrinsic capacity (IC) score, SPPB, MoCA and GDS-30 and log 10-transformed peak intensity of differential metabolites after adjusting for age and sex.

**Table S4.** Metabolite sets significantly enriched in each intrinsic capacity (IC) domain with fold enrichment > 1 and p-value < 0.05.

| **Domain of IC** | **Metabolite Set** | **Fold enrichment** | **Total** | **Hits** | **Metabolite** | | **Raw p-value** |
| --- | --- | --- | --- | --- | --- | --- | --- |
|  |  |  |  |  | **Up** | **Down** |  |
| Cognition | Carnitine Synthesis | 6.64 | 22 | 2 | Glycine; Succinic acid | — | 0.0343 |
|  | Glycine and Serine Metabolism | 3.72 | 59 | 3 | Glycine, L-Serine | Glyceric acid | 0.0420 |
| Psychology | Purine Metabolism | 5.93 | 74 | 3 | Hypoxanthine, Xanthine | Guanine | 0.0103 |
|  | Methylhistidine Metabolism | 36.63 | 4 | 1 | — | L-Histidine | 0.0271 |
| Sensory | Bile Acid Biosynthesis | 6.31 | 65 | 2 | — | Deoxycholic acid glycine conjugate, Taurodeoxycholic acid | 0.0350 |

Abbreviations: IC, intrinsic capacity.

**Table S5.** Over-represented pathways related to each intrinsic capacity (IC) domain with raw p-value < 0.05.

| **Domain of IC** | **Pathway** | **Match status** | **Metabolite** | | **Raw p-value** | **FDR** | **Impact** |
| --- | --- | --- | --- | --- | --- | --- | --- |
|  |  |  | **Up** | **Down** |  |  |  |
| IC | Pentose phosphate pathway | 2/22 | Gluconic acid | Glyceric acid | 0.0051 | 0.430 | 0.047 |
|  | Tryptophan metabolism | 2/41 | 5-Hydroxyindoleacetic acid, 5-Hydroxy-L-tryptophan | — | 0.0173 | 0.574 | 0.145 |
|  | D-Arginine and D-ornithine metabolism | 1/4 | — | 2-Oxoarginine | 0.0205 | 0.574 | 0.000 |
| Mobility | Arginine biosynthesis | 3/14 | Citrulline, Ornithine, Urea | — | 0.0041 | 0.345 | 0.289 |
|  | Glyoxylate and dicarboxylate metabolism | 3/32 | cis-Aconitic acid, Citric acid | Glyceric acid | 0.0413 | 1.000 | 0.135 |
| Cognition | Glyoxylate and dicarboxylate metabolism | 3/32 | L-Serine, Glycine | Glyceric acid | 0.0003 | 0.012 | 0.228 |
|  | Glycine, serine and threonine metabolism | 3/33 | L-Serine, Glycine | Glyceric acid | 0.0003 | 0.012 | 0.487 |
|  | Aminoacyl-tRNA biosynthesis | 2/48 | L-Serine, Glycine | — | 0.0179 | 0.302 | 0.167 |
|  | Phenylalanine, tyrosine and tryptophan biosynthesis | 1/4 | — | 4-Hydroxyphenylpyruvic acid | 0.0180 | 0.302 | 0.000 |
|  | D-Arginine and D-ornithine metabolism | 1/4 | — | 2-Oxoarginine | 0.0180 | 0.302 | 0.000 |
|  | Ubiquinone and other terpenoid-quinone biosynthesis | 1/9 | — | 4-Hydroxyphenylpyruvic acid | 0.0400 | 0.560 | 1.000 |
| Psychology | Purine metabolism | 3/65 | Hypoxanthine, Xanthine | Guanine | 0.0007 | 0.056 | 0.041 |
| Sensory | Glycerophospholipid metabolism | 2/36 | Phosphorylcholine | LysoPC(20:5) | 0.0031 | 0.257 | 0.027 |
|  | Valine, leucine and isoleucine biosynthesis | 1/8 | — | L-Leucine | 0.0205 | 0.861 | 0.000 |
|  | Butanoate metabolism | 1/15 | — | 2-Hydroxyglutarate | 0.0382 | 1.000 | 0.000 |

Abbreviations: IC, intrinsic capacity, FDR, false discovery rate.
